# Profiling hypoxia signaling reveals a lncRNA signature contributing to immunosuppression in high-grade glioma

**DOI:** 10.3389/fimmu.2024.1471388

**Published:** 2024-10-02

**Authors:** Xinqiao Li, Jingcheng Xu, Xue Li, Jianghua Shi, Chunmi Wei, Qingyu Liang

**Affiliations:** ^1^ Department of Neurosurgery, The First Hospital of China Medical University, Shenyang, Liaoning, China; ^2^ International Department, The First Hospital of China Medical University, Shenyang, Liaoning, China; ^3^ Department of Radiotherapy, The Affiliated Tumor Hospital of Guangxi Medical University, Nanning, Guangxi, China

**Keywords:** high-grade glioma, hypoxia, lncRNA, prognosis, immunotherapy

## Abstract

**Background:**

Hypoxic conditions in glioma are linked to tumor aggressiveness, poor prognosis, and treatment resistance. Long non-coding RNAs (lncRNAs) play key roles in the hypoxic and immune microenvironment of cancers, but their link to hypoxia-induced immunosuppression in high-grade glioma (HGG) is not well-studied.

**Methods:**

Gene expression profiles from TCGA and CGGA, along with clinical and genomic data, were analyzed. Bioinformatics methods including Consensus Clustering, Pearson correlation, and Cox regression analyses were used. Cell proliferation was assessed using cell counting kit-8 and colony formation assays. Glioma-macrophage interactions were evaluated using a co-culture model.

**Results:**

Hypoxia subtype clustering showed hypoxic stress correlates with worse HGG prognosis. Eight hypoxia-related lncRNAs (AP000695.4, OSMR-AS1, AC078883.3, RP11-545E17.3, LINC01057, LINC01503, TP73-AS1, and LINC00672) with prognostic value were identified, forming a risk signature that separated patients into distinct prognostic groups. Multivariate Cox regression confirmed the signature as an independent prognostic factor. High-risk patients had greater hypoxia, leading to an immunosuppressive environment and immunotherapy resistance via tumor-associated macrophages (TAMs). TP73-AS1 significantly influenced hypoxia-induced TAM infiltration and M2 polarization.

**Conclusions:**

We profiled hypoxic stress in HGG and developed an 8-lncRNA hypoxia-related signature predicting patient survival and immunotherapy response, emphasizing its role in hypoxia-induced immunosuppression.

## Introduction

High-grade glioma, the most prevalent and aggressive primary brain tumor in adults, has a poor prognosis despite advancements in treatment options such as surgery, radiotherapy, chemotherapy, and TTFields (1). This poor outcome is primarily due to extensive intratumoral heterogeneity, driven by diverse gene expression and varied tumor microenvironments, including hypoxia and immunosuppression (2–4). Glioma cells in hypoxic areas show greater resistance to radiotherapy and chemotherapy (5, 6).

Hypoxia, a key metabolic element of the tumor microenvironment (TME), contributes significantly to malignant progression. Responses to hypoxia within the TME are mainly mediated by hypoxia-inducible factors (HIF), which include HIF1, HIF2, and HIF3. These transcription factors stabilize in low oxygen conditions. During hypoxia, reduced activity of α-ketoglutarate-dependent dioxygenases and prolyl hydroxylases leads to HIFα stabilization and dimerization with the ARNT protein. The HIFα-ARNT complex then translocates to the nucleus, binding to hypoxia response elements in the promoter regions of target genes (7). Recent research indicates that hypoxia signaling can reshape the tumor’s immunosuppressive environment, affecting the response to immunotherapy (7). Thus, modulating hypoxia signaling could be a viable approach to overcoming immunotherapeutic resistance (8). However, the intricate relationship among hypoxia, the tumor’s immunosuppressive microenvironment, and immunotherapeutic resistance in high-grade gliomas (HGG) requires further exploration.

In recent years, significant progress has been made in understanding the role of long non-coding RNAs (lncRNAs) as critical regulators of tumor malignancy, including influencing pro-tumor environments like hypoxia (9) and immunosuppression (10), suggesting their potential as therapeutic targets (11). Several lncRNAs have been found to regulate hypoxia signaling and the immune microenvironment in HGG. For instance, blocking lncRNA HIF1A-AS2 hampers glioma cells’ adaptation to hypoxia by reducing HMGA1 expression (12). Huang et al. (13) found that high LUCAT1 expression enhances HIF1α activity by forming a complex with HIF1α and its co-activator CBP, regulating HIF1α target gene expression, which aids glioma stem-like cells in adapting to hypoxia. Silencing lncRNA NEAT1 inhibits M1 macrophage polarization and reduces the expression of TNFα and other inflammatory cytokines, improving the response to anti-PD-1/PD-L1 therapy in GBM (14). Despite these findings, a comprehensive understanding of how lncRNAs contribute to hypoxia-induced immunosuppression in HGG is still lacking. Thus, there is an urgent need to identify hypoxia-related lncRNAs and elucidate their mechanisms.

In this study, we analyzed public HGG transcriptomic data from The Cancer Genome Atlas (TCGA) and the Chinese Glioma Genome Atlas (CGGA). We first identified hypoxia-related lncRNAs through univariate Cox, Pearson correlation, clustering, and differential expression analyses. The hypoxia-related lncRNA signature was found to independently predict prognosis in HGG and enhance hypoxia signaling to reprogram the immune microenvironment. These findings contribute to a better understanding of hypoxia-induced immunosuppression and underscore the potential application of these eight hypoxia-related lncRNAs in HGG treatment strategies.

## Materials and methods

### Ethics statement

The ethical committee of The First Hospital of China Medical University approved the biological experiments conducted in this study (Ethics number: AF-SOP-07-1.2-01).

### Patient datasets and clinical information

LncRNA expression data for HGG patients was obtained from The Atlas of Noncoding RNAs in Cancer (TANRIC) (15), incorporating data from the TCGA glioma RNA-seq database (http://cancergenome.nih.gov/) and independent RNA-seq studies in the CGGA database (16). Coding mRNA expression profiles were sourced from the TCGA and CGGA-325 datasets (http://www.cgga.org.cn). Sample annotations were performed using barcode IDs based on clinical information from the UCSC Xena (https://xenabrowser.net/datapages/) and CGGA databases. Detailed clinical and molecular information is provided in **Supplementary Table S1**.

### Hypoxia subtypes clustering

Fifteen clinically relevant hypoxia-related metagenes (HRMG, **Supplementary Table S2**) were curated from literature reviews (17). To identify hypoxia subtypes within the TCGA-HGG cohort, the expression matrix of these metagenes was used with the Consensus Cluster Plus R package, utilizing Euclidean distance and K-means clustering. Cluster-consensus, delta area from unsupervised consensus clustering, and average silhouette width from the Silhouette R package were employed to validate clustering stability.

### Establishing the hypoxia-related lncRNA signature

To develop the hypoxia-related lncRNA signature, we utilized lncRNA expression profiles from TCGA RNAseq as a training set. Differentially expressed lncRNAs (|logFC| > 1, FDR < 0.001) were identified in comparisons of hypoxia clusters (cluster 1 vs. cluster 3, cluster 1 vs. cluster 4, cluster 2 vs. cluster 3, cluster 2 vs. cluster 4) using the Limma package as descibed in the previous study (18). Prognosis-related lncRNAs (p-value < 0.05) were identified via univariate Cox regression analyses of HGG, WHO grade 3, and WHO grade 4 glioma cohorts from TCGA RNAseq. Pearson correlation analysis (|Pearson r| > 0.5, FDR < 0.001) was used to correlate lncRNAs with hypoxia metagenes. Eight lncRNAs were identified by overlapping the above five correlated gene lists to construct a hypoxia-related lncRNA signature. Risk scores were computed by linearly aggregating the expression values of these lncRNAs weighted by coefficients from univariate Cox regression analysis in TCGA. These coefficients were also applied to calculate risk scores in the validation dataset (CGGA).

### Screening signature-related genes in HGG

Pearson’s correlation analysis was used to determine the relationship between coding mRNA expression and risk scores in HGG. Genes with Pearson r > 0.7 and p-value < 0.05 were selected for Gene Ontology (GO) analysis using the ClueGO plug-in in Cytoscape.

### Calculation of scores on gene sets and biological features

Scores for gene sets, including HRMG, immunosuppressive gene sets, immune-cell-related gene sets, and gene programs and pathway signatures (19–21), were calculated using ssGSEA. The stromal score, immune score, and tumor purity were calculated using the ESTIMATE R package (22).

### Cell lines and cell culture

Human glioma cell lines LN229 and U87 were procured from the American Type Culture Collection (ATCC, Manassas, VA, USA). Initially, cells were cultured in Dulbecco’s modified Eagle’s medium (DMEM, Gibco) supplemented with 10% fetal bovine serum (Gibco). Upon reaching logarithmic growth phase, cells were enzymatically dissociated into single cells using 0.25% trypsin (Gibco). All cell lines underwent fewer than 20 passages and passed mycoplasma and short tandem repeat (STR) DNA profiling tests.

### Quantitative real-time PCR

Total RNA was extracted using TRIzol (TaKaRa, Japan) as per the manufacturer’s instructions. Subsequently, RNA was reverse-transcribed into cDNA using PrimeScript™ RT Master Mix (RR036A, Takara). Quantitative PCR was conducted with TB Green^®^ Premix Ex Taq™ (RR420A, Takara) using a LightCyclerR480 (Roche Diagnostics Ltd., Basel, Switzerland) under identical amplification conditions. Each reaction was performed in triplicate. Gene expression levels were normalized to 18S and quantified using the 2^-△△Ct^ method. The primer sequences were as follows: TP73-AS1 forward, 5’-CTCCGGACACTGTGTTTTCTC-3’; TP73-AS1 reverse, 5’-TCTTTTAAGGCGGCCATATC-3’; 18S forward, 5’-GCAGAATCCACGCCAGTACAAGAT-3’; 18S reverse, 5’-TCTTCTTCAGTCGCTCCAGGTCTT-3’.

### Western blotting

Proteins from glioma cell lines were extracted using RIPA lysis buffer with PMSF. Protein concentration was determined using the BCA method, and 15-30 µg of protein was loaded per well and separated by SDS-PAGE. Proteins were transferred onto PVDF membranes (0.22 μm pore size) and incubated with primary antibodies against GAPDH (1:5000; 10494-1-AP; ProteinTech) and HIF-1A (1:2000; 20960-1-AP; ProteinTech). After incubation with secondary antibody (ProteinTech), signals were detected using the chemiluminescence Femto-sig ECL kit (Tanon, Shanghai, China).

### Immunohistochemical staining

Consent was obtained from all glioma patients, and the use of human samples for IHC was approved by the Institutional Review Board of The First Affiliated Hospital of China Medical University. Brain tumor tissues were fixed in 4% paraformaldehyde, paraffin-embedded, and sectioned into 4 µm slices. Sections were incubated with primary antibodies against IBA-1 (1:500; 10904-1-AP, Proteintech), HIF-1A (1:500; 20960-1-AP, Proteintech), and TGF-β (1:500; 21898-1-AP, Proteintech). Imaging was performed using an optical microscope (Olympus, Tokyo, Japan), and German immunohistochemical staining intensity was applied.

### Flow cytometry analysis

THP-1 cells were differentiated into macrophages by treatment with 100 nM phorbol 12-myristate 13-acetate (PMA; Sigma) for 24 hours. After differentiation, 2×10^5^ THP-1 cells were seeded into the lower chamber of a 24-well plate in RPMI-1640 medium supplemented with 10% FBS. LN229 cells were seeded at a density of 1×10^5^ cells per well in the upper chamber of a co-culture insert with a 3 µm pore polycarbonate membrane (Corning, Corning, NY, USA) in medium under different conditions (si-NC + DMSO (control), si-NC + CoCl_2_, si-TP73-AS1 + CoCl_2_). Co-culture was maintained for 48 hours to allow interaction between LN229 and THP-1 cells. Following co-culture, transwell inserts were removed, THP-1 cells from the lower chamber were collected, and PE-CD206 (PE-65155, Proteintech), FITC-MHC II (FITC-65218, Proteintech), and APC-CD11b (101211, BioLegend) flow cytometry antibodies were then added and incubated at 4°C for 30 min while in the dark. The unbound antibodies were cleaned with pre-cooled PBS after centrifugation, and the supernatant was then discarded. The fluorescence values of the macrophage differentiation antigen on cell surface were measured using a flow cytometer LS Fortessa (BD Biosciences).

### Fluorescence *in situ* hybridization

FISH was performed to visualize the location of TP73-AS1 in glioma using Cy3-labeled probes synthesized by Servicebio (Wuhan, China). Hybridization was conducted overnight following the manufacturer’s instructions, and images were captured using a laser scanning confocal microscope (Olympus, Tokyo, Japan).

### Immunofluorescence

Clinical glioma tissue samples were fixed in 4% paraformaldehyde, embedded in paraffin, and sectioned at 4 µm. Sections were processed by Wuhan Servicebio Technology Co., Ltd. for multicolor immunofluorescence staining. After antigen retrieval and blocking, primary antibodies against HIF-1α (green; 20960-1-AP, Proteintech) and IBA-1 (yellow; 10904-1-AP, Proteintech) were applied overnight at 4°C. Following washing, sections were incubated with Alexa Fluor 488-conjugated anti-rabbit IgG and Alexa Fluor 594-conjugated anti-mouse IgG secondary antibodies. FISH staining for TP73-AS1 (red) was conducted using a commercial kit, and nuclei were stained with DAPI (blue). Images were acquired using a microscope (Olympus).

### Correlation calculation

In FISH and IHC staining results, three fields of view from the same specimen were selected for positive cell counting, and mean values were calculated. Correlation analysis was performed using GraphPad, and regression curves were plotted.

### Construction of a cellular hypoxia model

To induce hypoxia, CoCl_2_ was used as a hypoxia-mimetic agent (23). LN229 and U87 cells were seeded at a density of 5×10^5^ cells per well in 6-well plates and allowed to adhere overnight. The next day, medium was replaced with DMEM containing 100 µM CoCl_2_ (Macklin, Shanghai). Cells were exposed to CoCl_2_ for varying duration (6, 12, and 36 hours) to assess hypoxia induction. Post-treatment, cells were washed with PBS, and HIF-1α levels were evaluated by Western blotting to confirm hypoxia induction.

### Cell transfection

siRNAs targeting TP73-AS1 were synthesized by Ribobio (Guangzhou, China) with the following sequences: si-TP73-AS1-1 sense, 5’-GTTTCCTGCTTTCCAAGTAAA-3’; si-TP73-AS1-2 sense, 5’-CGGTGTAAATTGACTCAGAAA-3’; and si-TP73-AS1-3 sense, 5’-CTTTCCTTTAACTCAAGTAAA-3’. Suppression of TP73-AS1 was validated using qRT-PCR.

### Colony formation assay

LN229 glioma cells were transfected with siRNA negative control (si-NC) or siRNAs targeting TP73-AS1 (si-TP73-AS1-1 and si-TP73-AS1-2). After 48 hours, transfected cells were trypsinized, counted, and seeded in 12-well plates at a density of 500 cells per well. Cells were cultured in DMEM supplemented with 10% fetal bovine serum for 14 days to allow colony formation. Colonies were fixed with 4% paraformaldehyde, stained with 0.1% crystal violet, and counted. The experiment was performed in triplicate to assess the effect of TP73-AS1 knockdown on LN229 cell clonogenic potential.

### Cell viability assay

Cell viability of glioma cells was assessed using a Cell Counting Kit-8 (Beyotime) according to the manufacturer’s protocol. Glioma cells were seeded in triplicate in 96-well plates at a density of 1×10^3^ cells/well and incubated for various duration (0, 24, 48, 72, 96, and 120 hours). Cell viability was measured using a microplate reader (BioTek).

### TAM migration assay

THP-1 cells were differentiated into macrophages by treatment with 100 nM PMA (Sigma) for 24 hours. Following differentiation, 1×10^5^ THP-1 cells were seeded in the upper chamber of a transwell insert with an 8 µm pore membrane (Corning) in RPMI-1640 medium supplemented with 10% FBS. LN229 cells were seeded at a density of 2×10^5^ cells per well in the lower chamber of a 24-well plate under different conditions (si-NC + DMSO (control), si-NC + CoCl_2_, si-TP73-AS1 + CoCl_2_). Co-culture was maintained for 48 hours to allow interaction between LN229 and THP-1 cells.

### Statistical analysis

Statistical analyses were primarily conducted using GraphPad Prism 8.0.2 and R v3.5.0 (http://www.R-project.org) software. Group differences were assessed using Wilcoxon rank-sum test, Welch t-test, one-way ANOVA, or chi-square test. Kaplan-Meier survival curves were estimated using the log-rank test. Univariate and multivariate Cox regression analyses identified prognostic factors. Pearson correlation analysis determined associations between continuous variables. All experiments underwent at least three independent replications and were presented using mean ± standard deviation. A two-sided p-value < 0.05 was considered statistically significant unless otherwise stated. Benjamini and Hochberg (BH or FDR) correction was applied to reduce false positive rates.

## Results

### Hypoxia status could distinguish the prognosis of HGG

To explore the prognostic value of hypoxia status in HGG (Grade 3/4), we conducted an unsupervised consensus cluster analysis of HGG samples from TCGA based on the gene expression profiling of a common HRMG (**Supplementary Figure S1**). According to the cluster consensus, delta area, and average silhouette width, four robust hypoxia clusters were established for HGG samples (**Supplementary Figures S2A–D**). We observed that different hypoxia clusters yielded distinct survival prognosis (**Supplementary Figure S2E**). Tumors stratified into Cluster 1 and 2 were closely correlated with worse prognosis. Taken together, the status of hypoxia status may significantly impact clinical prognosis.

### Identification of eight hypoxia-related lncRNAs in patients with HGG

**Figure 1:**
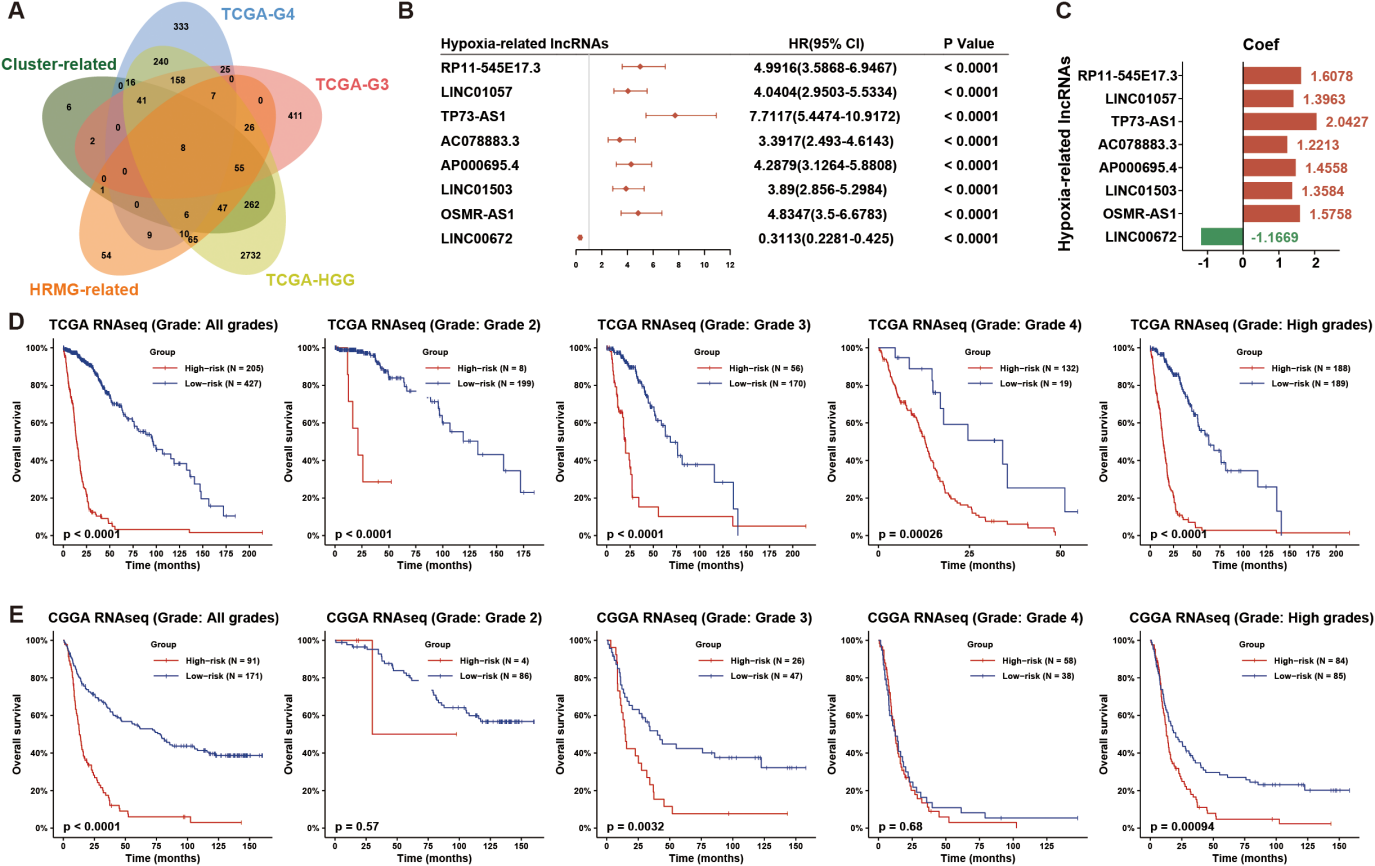
Eight hypoxia-associated lncRNAs identified in HGG patients. **(A)** The Venn diagram illustrates the hypoxia-related lncRNAs discovered by overlapping five lists: prognosis-related lncRNAs in HGG, WHO grade 3 (G3) glioma, WHO grade 4 (G4) glioma, hypoxia-cluster-associated lncRNAs, and HRMG-related lncRNAs from the TCGA dataset. **(B)** A forest plot shows univariate Cox regression analysis results for the 8 hypoxia-related lncRNAs in HGG from the TCGA dataset. **(C)** Coefficients for these 8 hypoxia-related lncRNAs were obtained through univariate Cox regression analysis in HGG from the TCGA dataset. **(D, E)** The hypoxia-related 8-lncRNA risk signature demonstrated significant prognostic value across all grades, each grade, and HGG in both TCGA and CGGA datasets. HGG, high-grade glioma; HRMG, hypoxia-related metagenes.

To further explore the underlying molecules involved in HGG hypoxia status, we conducted univariate Cox analyses on lncRNAs in HGG, WHO grade 3 (G3) glioma, and WHO grade 4 (G4) glioma cohorts from TCGA set. Meanwhile, we performed the Pearson correlation analysis between lncRNAs and HRMG, and conducted differential lncRNA expression analysis across hypoxia clusters. We overlapped the above 5 correlated lncRNA lists (Cox analysis: p-value < 0.05; Pearson correlation analysis: |Pearson r| >0.5, FDR < 0.001; differential expression analysis: |logFC| >1, FDR < 0.001) and obtained eight hypoxia-related lncRNAs (**Figures 1A, B**): AP000695.4, OSMR-AS1, AC078883.3, RP11-545E17.3, LINC01057, LINC01503, TP73-AS1 and LINC00672.

To evaluate the prognosis of glioma patients by hypoxia-related lncRNAs, we calculated the risk score based on coefficients from Cox analysis and lncRNA expression for each of the TCGA and CGGA glioma samples (**Figure 1C**). To analyze the prognosis of the hypoxia-related 8-lncRNA risk signature, we dichotomized TCGA and CGGA glioma samples based on the median risk score. The overall survival (OS) time differed significantly between the low- and high-risk groups (**Figures 1D, E**). Low risk score resulted in a significantly favorable prognosis than high risk score. Taken together, we developed a hypoxia-related lncRNA signature with robust prognostic value in HGG.

### Hypoxia-related lncRNA risk signature showed specific clinical and molecular features in HGG

**Figure 2:**
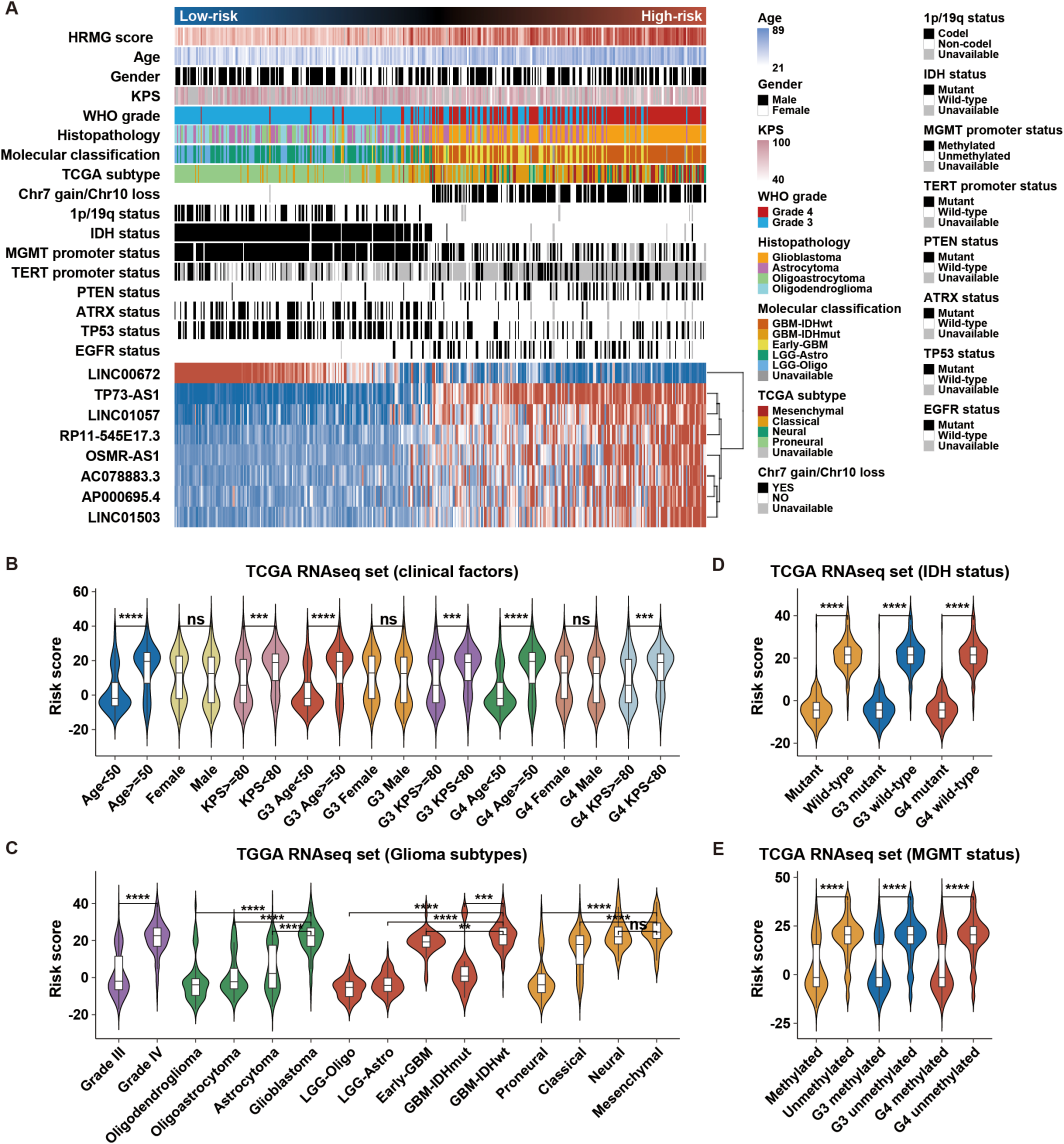
Association of the hypoxia-related risk signature with clinical and molecular characteristics in TCGA. **(A)** Distribution of clinicopathological and molecular features correlated with the hypoxia-related 8-lncRNA signature, sorted by ascending risk scores in HGG. **(B–E)** Significant differences in risk scores were observed among clinical factors, glioma subtypes, IDH status, and MGMT status. **p < 0.01; ***p < 0.001; ****p < 0.0001; ns, not significant.

To analyze the relation between hypoxia-related lncRNA risk score and HGG clinical and molecular features, we drew heatmaps (**Figure 2A**; **Supplementary Figure S3A**) to show the spectrums of clinical and molecular features based on risk score. There were robust differences in the clinical and molecular characteristics of the two risk groups (**Figure 2A**, **Supplementary Figure S3A**, **Supplementary Table S1**). High-risk groups were closely correlated with older age at diagnosis, higher Karnofsky performance score (KPS), higher WHO grade, glioblastoma (GBM) phenotype, classical or mesenchymal subtype, Chr 7 gain/Chr 10 loss, non-codeleted 1p/19q, wild type IDH, unmethylated MGMT promoter, mutant TERT promoter, mutant PTEN, wild type ATRX, wild type TP53, and mutant EGFR. We also explored the association between risk score and various clinical, pathological, and molecular features. Higher risk score was observed in HGG with risky clinical factors and subtypes (**Figures 2B, C**; **Supplementary Figure S3B**). HGG with wild-type IDH, unmethylated MGMT promoter, and non-codeleted 1p/19q presented higher risk score (**Figures 2D, E**; **Supplementary Figure S3B**). In summary, these results indicated that the hypoxia-related lncRNA risk signature was positively associated with risky behaviors in HGG.

### Hypoxia-related lncRNA risk signature exhibited significant prognostic value in various stratified cohorts of HGG

**Figure 3:**
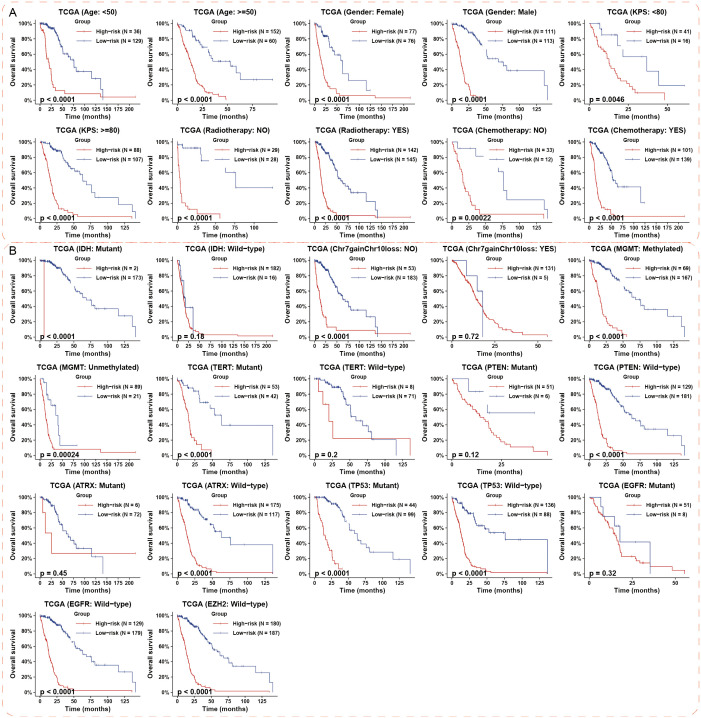
Prognostic impact of the hypoxia-associated 8-lncRNA risk signature in different HGG subgroups. **(A)** The risk signature demonstrated significant prognostic value across various cohorts stratified by clinical features in the TCGA datasets. **(B)** The prognostic value of the risk signature was most notable in subgroups defined by key molecular events in TCGA datasets. p-values were calculated using the log-rank test for trend. HGG, high-grade glioma.

**Figure 4:**
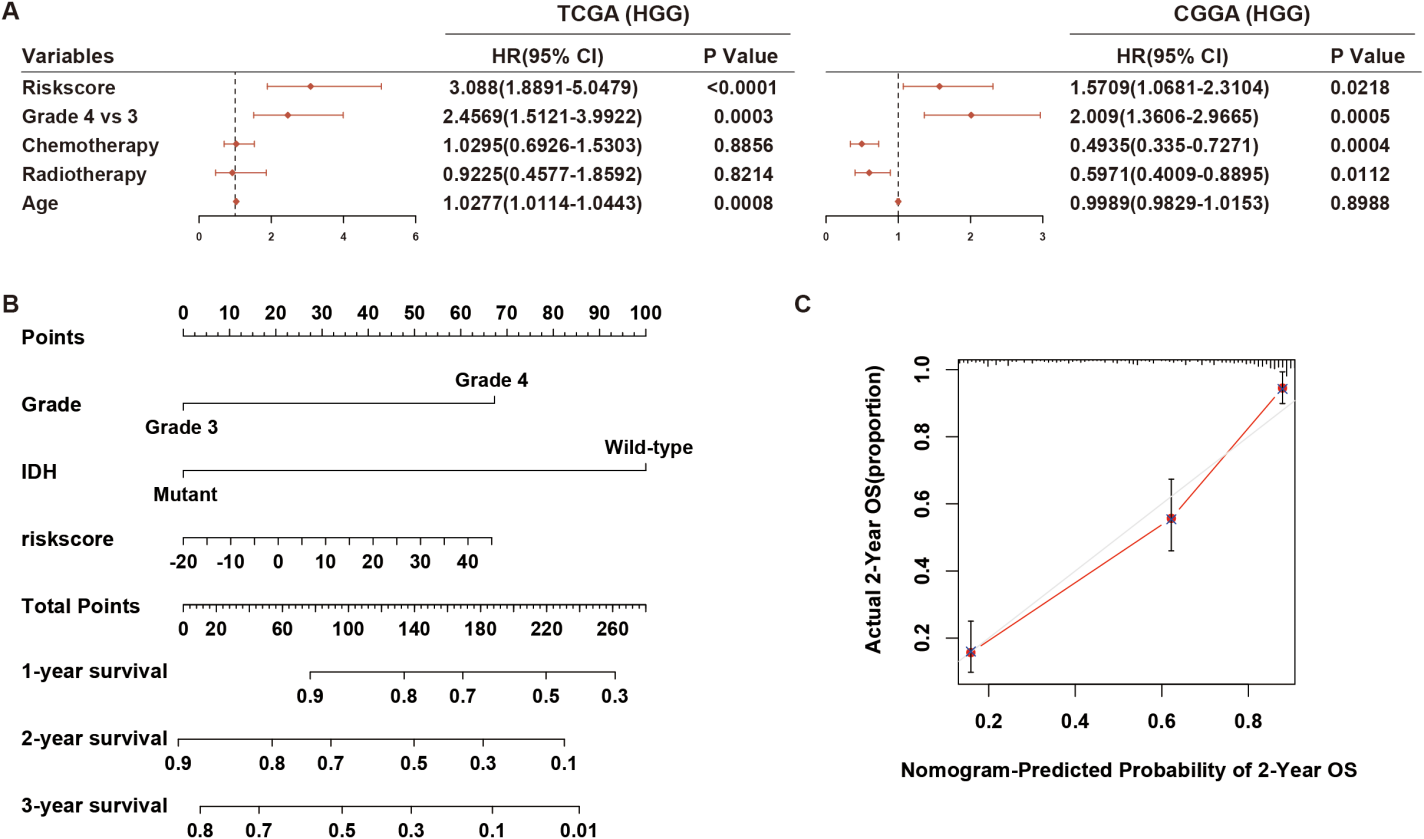
Multivariate Cox regression analysis and prognostic prediction of the risk signature. **(A)** The forest plot illustrates the independent prognostic value of the risk signature compared to WHO grade, chemotherapy, and radiotherapy in HGG cohorts from TCGA and CGGA datasets. **(B, C)** Nomogram plots and calibration curves based on IDH status, WHO grade, and the lncRNA risk score were developed to illustrate the prognostic prediction of the risk signature in HGG cohorts from the TCGA dataset. HGG, high-grade glioma.

To precisely predict the prognosis of HGG, we examined the prognostic significance of the hypoxia-related 8-lncRNA risk signature across various stratified cohorts. Within the TCGA and CGGA dataset, our analysis demonstrated that the risk score had substantial predictive value in groups categorized by age, gender and KPS (**Figure 3A**; **Supplementary Figure S4**). Additionally, high-risk groups had a shorter survival time than low-risk groups, irrespective of whether patients underwent radiotherapy or chemotherapy (**Figure 3A**; **Supplementary Figure S4**). Then, we evaluated the prognostic significance of the risk score across various molecularly stratified cohorts. In cases with different statuses of MGMT promoter and TP53, those with high risk score exhibited shorter survival times compared to those with low risk score (**Figure 3B**). High risk score was associated with reduced OS time in cases without Chr 7 gain/Chr 10 loss and with mutation of IDH and TERT promoter and wild-type of PTEN, ATRX, EGFR and EZH2 in the TCGA dataset (**Figure 3B**), which was similar in the CGGA dataset (**Supplementary Figure S4**). Furthermore, multivariate Cox analysis indicated that the risk signature was an independent prognostic factor in HGG (**Figure 4A**). Finally, the nomogram plots and calibration curves based on IDH status, WHO grade, and the lncRNA risk score suggested that the risk signature could increase prediction accuracy of prognosis in HGG (**Figure 4B**; **Supplementary Figure S5**). Overall, the risk signature proved to be a significant predictor of prognosis in HGG.

### HGG with high risk score exhibited immunosuppressive phenotypes

**Figure 5:**
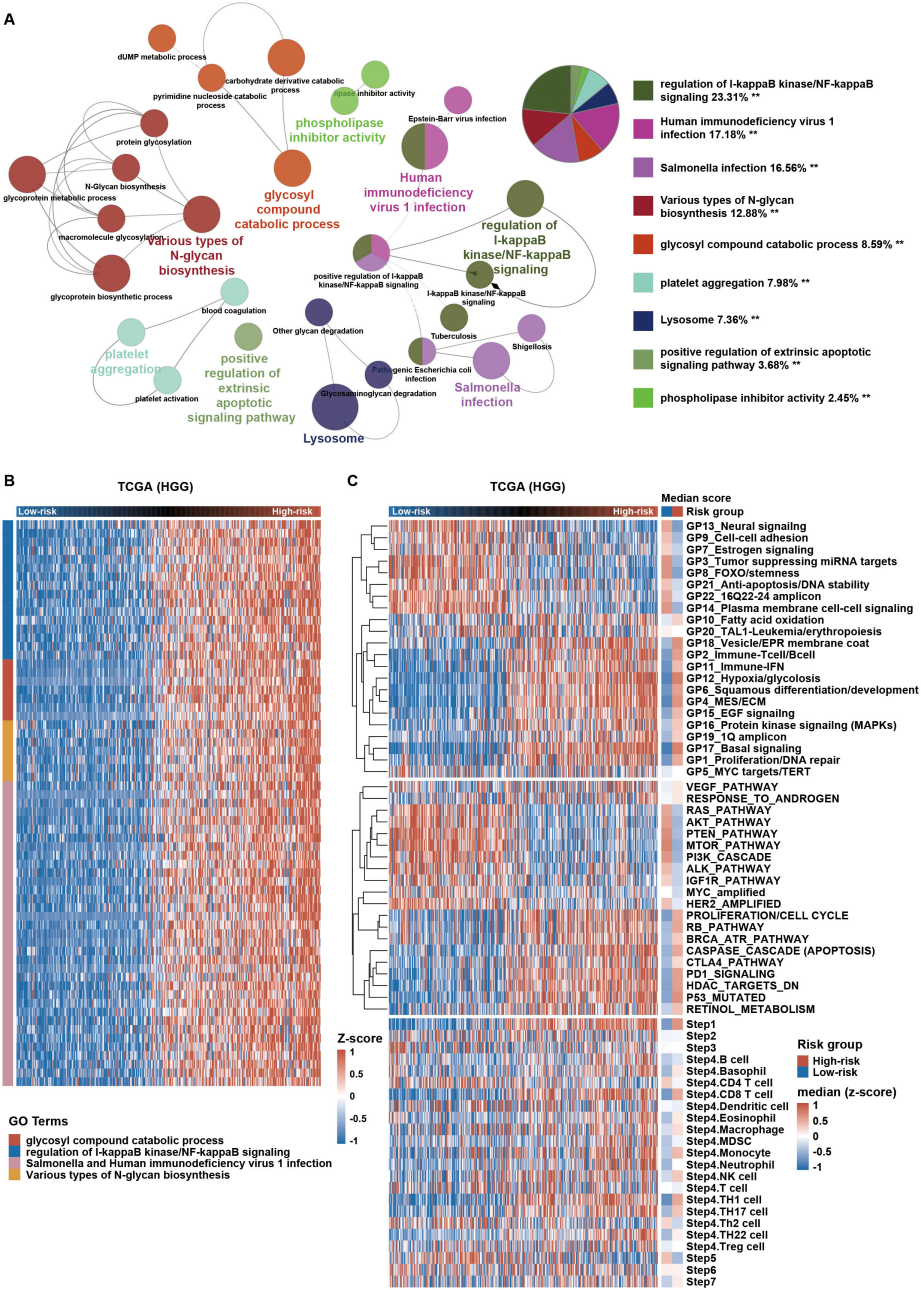
High-risk HGG shows immunosuppressive characteristics. **(A)** Gene Ontology (GO) analysis using the ClueGO plugin in Cytoscape software was conducted for coding genes positively correlated with the risk score in TCGA. **p < 0.01. **(B)** Expression profiles of genes enriched in relevant GO terms are displayed in a heatmap across HGG. **(C)** Distribution of gene set scores from gene programs, pathway signatures, and tumor immunophenotype (TIP; http://biocc.hrbmu.edu.cn/TIP/) sorted by ascending risk scores across HGG. HGG, high-grade glioma.

**Figure 6:**
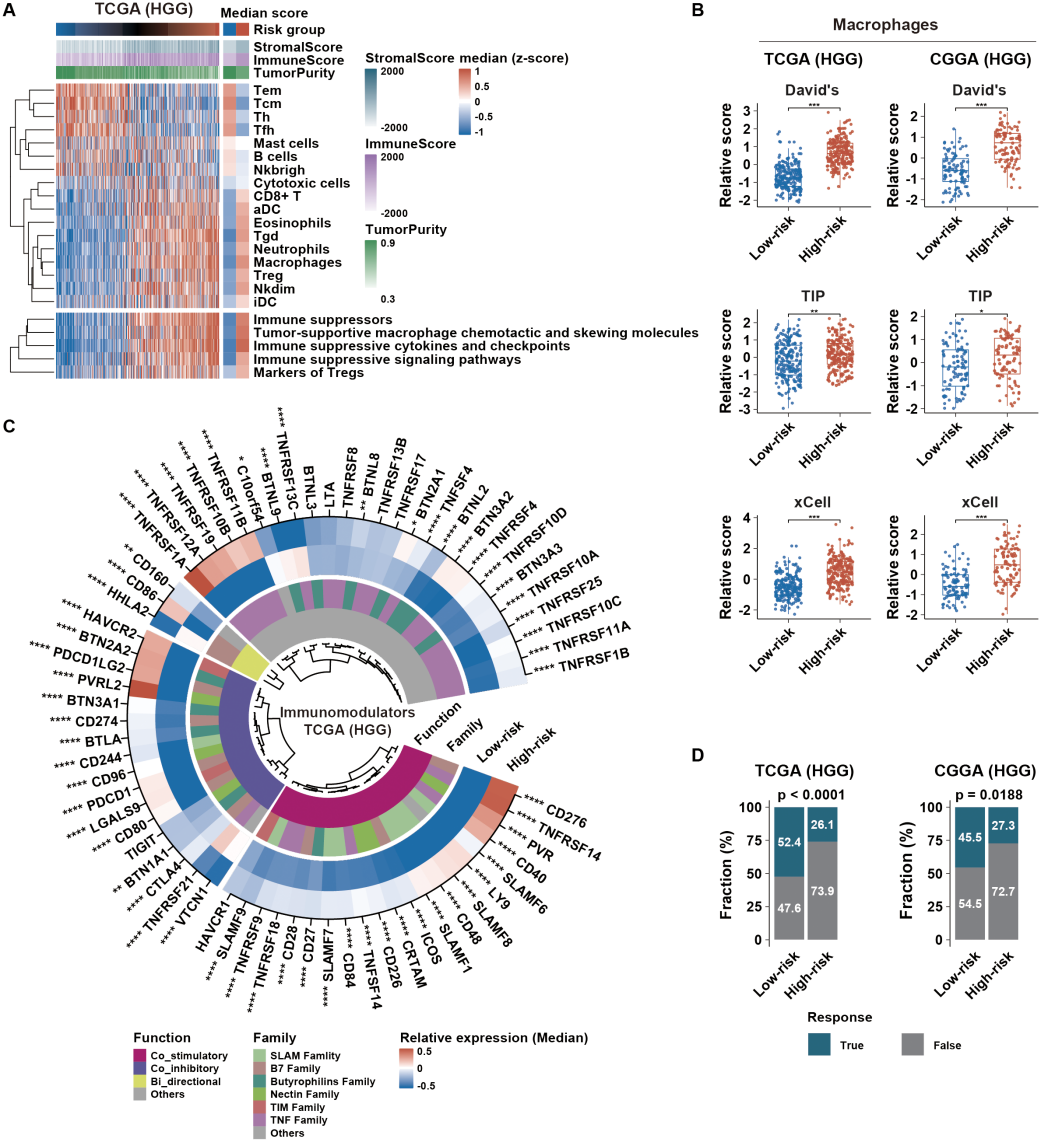
Immune microenvironment and immunotherapy response in different HGG risk groups. **(A)** Distribution of tumor immune microenvironment-related scores, sorted by ascending risk scores across HGG. The left heatmap indicates the median distribution of related scores in each risk group. **(B)** Distribution of macrophage scores calculated from different gene sets across risk groups. **(C)** Immunomodulator profile differences between risk groups were assessed using the Wilcoxon test. Color intensities represent the median relative expression of each immunomodulator in each risk group. **(D)** Glioma immunotherapy responsiveness predicted using the TIDE algorithm across risk groups in TCGA and CGGA datasets. p-values were calculated using the chi-square test. *p < 0.05; **p < 0.01; ***p < 0.001; ****p < 0.0001; HGG, high-grade glioma; TIDE,Tumor Immune Dysfunction and Exclusion.

To explore the biological phenotype of HGG cases between the high- and low-risk groups, we first selected the coding genes that positively correlated with the risk score (Pearson r > 0.7, p-value < 0.05) in HGG from TCGA dataset. These coding genes was functionally annotated by the ClueGO plugin in Cytoscape (**Figure 5A**). High-risk group were mainly associated with increased expression of regulation of I-kappaB kinase/NF-kappaB signaling, glycosyl compound catabolic process, Various types of N-glycan biosynthesis, Salmonella and Human immunodeficiency virus 1 infection (**Figure 5B**). Specifically, we analyzed the distribution of gene score calculated from nonredundant gene programs, pathway signatures for drug targets, and canonical pathways across various risk groups. We found that Hypoxia/glycolosis gene set and immune-related gene sets, such as GP2_Immune-T cell/B cell, GP11_Immune-IFN, PD1_signaling, and CTLA4_pathway (**Figure 5C**; **Supplementary Figure S6**), were consistently more enriched in high-risk group compared to low-risk group. We then analyzed the anti-tumor immunity cycle using the Tumor Immunophenotype (TIP) algorithm (19). Effective anti-tumor immunity was reduced at steps 6 (tumor cell recognition by T cells) and 7 (killing of cancer cells) in high-risk group (**Figure 5C**; **Supplementary Figure S6**). Additionally, high-risk group also had higher immunosuppressive score as determined by a previously described method (24) (**Figure 6A** lower panel and **Supplementary Figure S7A** lower panel). Collectively, these findings indicate that HGG with high risk score possess an immunosuppressive phenotype.

### Potential mechanisms of the immunosuppressive phenotype in HGG with high risk score

According to immunoediting theory, tumor immune microenvironment was remodeled by external and internal factors (25). We first explored potential external factors by investigating the microenvironmental composition. Our findings showed that high-risk group had elevated immune score and reduced tumor purity compared to low-risk group (**Figure 6A**; **Supplementary Figure S7A**). Furthermore, macrophages, Tregs, Neutrophils, Nkdim, iDC, Tgd, Esoinophils, aDC and CD8^+^ T cells were more prevalent in high-risk group. Based on the CIBERSORT algorithm, we found that macrophages were the predominant cells in HGG (**Supplementary Figure S7B**). High-risk group had higher macrophage score generated by single-sample ssGSEA algorithm based on three established gene sets (**Figure 6B**). Additionally, higher proportion of M2 macrophages was observed in high-risk group (**Supplementary Figure S7B**). We then concentrated on the expression of immunomodulators, including both costimulatory and coinhibitory molecules, as a significant factor in intrinsic immune escape regulation. In high-risk group, these molecules were notably upregulated (**Figure 6C**; **Supplementary Figure S7C**), indicating cytotoxic T-cell dysfunction. Subsequently, prediction analysis of glioma immunotherapy responsiveness based on the TIDE algorithm revealed that HGG with high risk score exhibited a poorer response to immunotherapy (**Figure 6D**). Therefore, enrichment of M2 macrophages and aberrant expression patterns of immunomodulators might serve as external and internal factors in maintaining immunosuppression in HGG with high risk score, resulting in immunotherapy resistance.

### TP73-AS1 inhibition suppresses malignant biological behaviors and hypoxia-induced TAM infiltration in HGG

**Figure 7:**
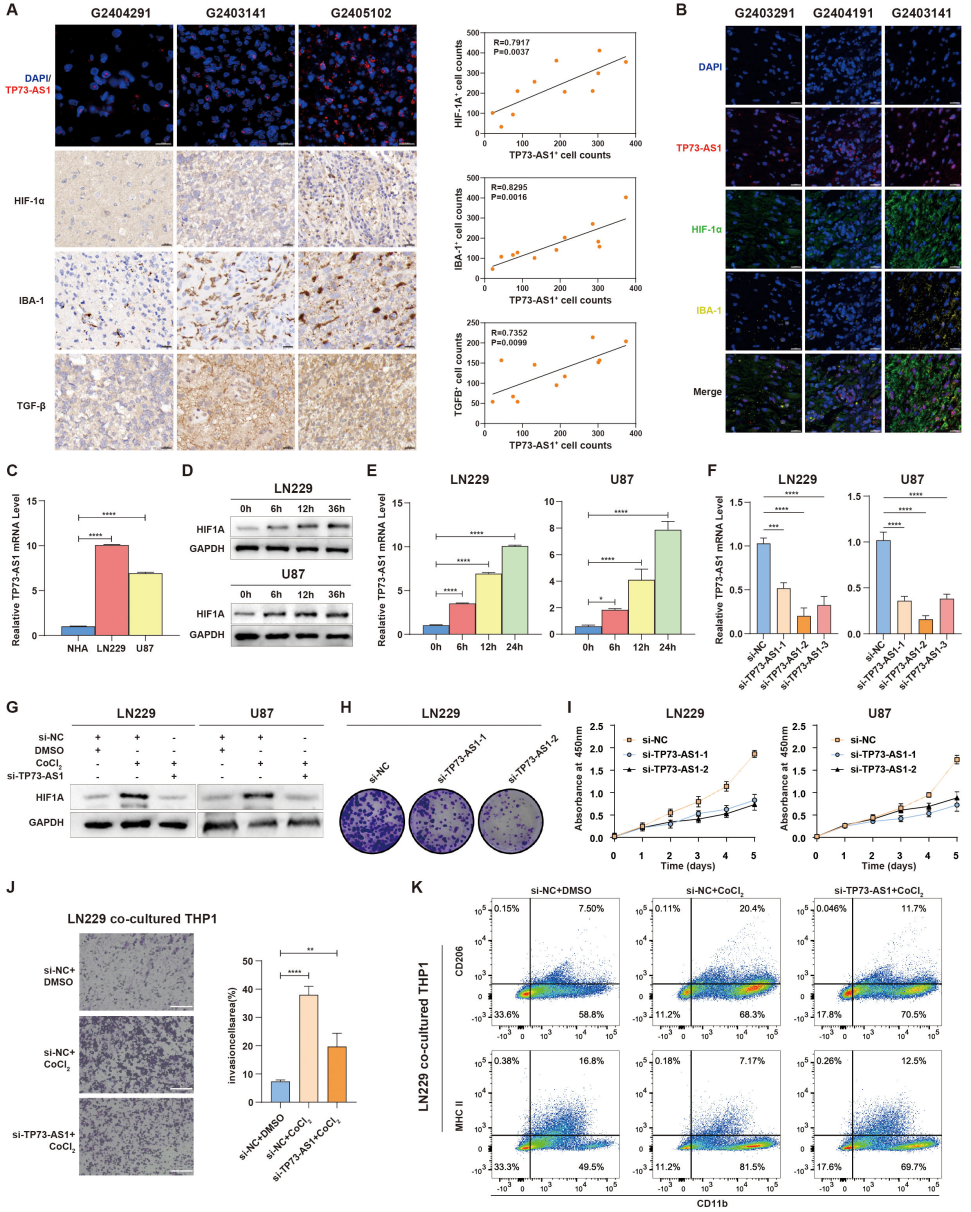
TP73-AS1’s role in HGG cell malignancy and interaction with TAM. **(A)** Fluorescence *in situ* hybridization of TP73-AS1, immunohistochemistry of HIF1α, TGF-β, IBA-1, and quantitative correlation analyses of HIF1α expression, TGF-β expression, IBA-1^+^ TAM infiltration, and TP73-AS1 expression in glioma patients. Scale bar: 20 μm. **(B)** Multicolor immunofluorescence staining examined the relationship between TP73-AS1, HIF1α, and IBA-1 in glioma patients. Scale bar: 20 μm. **(C)** TP73-AS1 expression in NHA, LN229, and U87 cells was measured by qRT-PCR. **(D)** Cellular hypoxia model validation by western blot analysis of HIF1α expression in LN229 and U87 at various time points. **(E)** qRT-PCR analysis of TP73-AS1 expression in LN229 and U87 over different time points under CoCl_2_-induced hypoxia condition. **(F)** Efficiency of si-TP73-AS1-1, -2, and -3 in LN229 and U87 glioma cells validated by qRT-PCR. **(G)** Western blot analysis of HIF1α expression in LN229 and U87 treated with CoCl_2_ or TP73-AS1 targeted siRNAs. **(H, I)** Cell proliferation after si-TP73-AS1-1 and -2 transfection, determined by colony formation and CCK-8 assays. **(J, K)** Migration analysis of THP-1 and flow cytometry of THP-1 polarization markers (M1: MHCII, M2: CD206), co-cultured with LN229 treated with DMSO, CoCl_2_, TP73-AS1 targeted siRNAs, or control siRNA. **p < 0.01; ***p < 0.001; ****p < 0.0001; DMSO, dimethyl sulfoxide.

To further validate the link between hypoxia-related lncRNAs and tumor immunosuppression, TP73-AS1 with highest expressional level in TCGA and CGGA compared to other hypoxia-related lncRNAs was selected as a representative lncRNA for this purpose (**Supplementary Figure S8**). In in-house glioma samples, we detected TP73-AS1, HIF1α, IBA-1, and TGF-β, which revealed that patients with elevated TP73-AS1 expression showed increased TAM (IBA-1^+^) infiltration and higher levels of HIF1a and TGF-β (**Figures 7A, B**), indicating a positive relationship between hypoxia-related TP73-AS1 and tumor immunosuppression. TP73-AS1 expressional levels were higher in HGG cell lines than in normal glial cells (**Figure 7C**). Under hypoxic conditions, HGG cells expressed more HIF1α and TP73-AS1, suggesting that hypoxia induces TP73-AS1 expression (**Figures 7D, E**). Moreover, TP73-AS1 knockdown (**Figure 7F**) inhibited HGG cell hypoxia status, proliferation, and colony formation (**Figures 7G–I**). To elucidate the role of hypoxia-related TP73-AS1 in hypoxia-induced TAM’s biological phenotype, we co-cultured HGG cells with suppressed TP73-AS1 with TAM under hypoxic conditions. The results showed that TP73-AS1 suppression in HGG cells significantly reduced hypoxia-induced TAM infiltration and M2 polarization (**Figures 7J, K**). These findings highlight the critical role of hypoxia-related lncRNA in regulating HGG’s malignant behaviors and TAM infiltration.

## Discussion

Hypoxia significantly influences both tumor development and immunosuppression within tumors (7). While most research on the hypoxic tumor microenvironment has concentrated on protein-coding genes (26), the role and impact of non-coding RNAs (ncRNAs) remain underexplored. Therefore, it is crucial to clarify the mechanisms and regulatory functions of hypoxia-related lncRNAs, a major component of ncRNAs, to better guide personalized HGG treatment. Our study examined the molecular features and biological functions of hypoxia-related lncRNAs in HGG across TCGA and CGGA datasets, with some findings validated through biological experiments. The research yielded three key findings: (1) the establishment of hypoxia-related 8-lncRNA risk signature, characterized by distinct molecular, biological, and clinical features; (2) the identification of a positive association between this risk signature and HGG immunosuppression; and (3) the demonstration that suppressing TP73-AS1, a representative hypoxia-related lncRNA, enhances the anti-tumor response in HGG cells.

The hypoxic microenvironment created by tumor cells can accelerate tumor progression. Numerous studies have indicated that lncRNAs play a crucial role in sustaining tumor cell homeostasis and enabling tumor cells to adapt and survive under hypoxic stress (9, 27). For instance, HIF1A-AS2 promotes the expression of HMGA1 through interactions with IGF2BP2 and DHX9, aiding glioblastoma stem-like cells (GSCs) in adapting to hypoxia within the tumor microenvironment (12). Similarly, the hypoxia-induced LUCAT1 forms a complex with HIF1α and its co-activator CBP, thereby regulating HIF1α target gene expression and GSC adaptation to hypoxia (13). In this study, we developed a hypoxia-related 8-lncRNA risk signature that is associated with prognosis in HGG. This risk signature also demonstrated strong predictive value. Additionally, the risk scores showed a positive correlation with risky behaviors and had significant predictive value for hypoxia status in HGG. Thus, a risk signature composed of only eight hypoxia-related lncRNAs proves to be effective for forecasting the prognosis of HGG patients.

Glioma cells do not operate in isolation but rather depend on the TME to evade host immunosurveillance. Hypoxia, as opposed to normoxia, is associated with increased immunosuppression due to a higher presence of TAMs (8, 28). Consequently, enhanced hypoxia in HGG may reprogram the immune microenvironment, leading to heightened tumor immunosuppression. This suggests that alleviating hypoxia could potentially improve immunotherapy outcomes in HGG. Hypoxia also upregulates tumor-associated lncRNAs and inhibits immune cell functions, facilitating tumor immune escape (29), underscoring the critical role of lncRNAs in hypoxia-induced immunosuppression. Currently, there is a lack of systematic research on the impact of these lncRNAs on hypoxia-induced immunosuppression in HGG. In this study, we further examined the biological functions associated with the hypoxia-related lncRNA signature. The high-risk group exhibited enrichment in the activation of I-kappaB kinase/NF-kappaB signaling, glycosyl compound catabolic process, and Various types of N-glycan biosynthesis, which are characteristic of tumor biology under hypoxic conditions (26). Additionally, the hypoxia-related lncRNA signature significantly correlated with the immune status in HGG. Risk scores were positively associated with the enrichment of pro-tumor immune cells in the HGG microenvironment, particularly M2 macrophages, indicating reduced sensitivity to immunotherapy. Despite the widespread use of immune checkpoint blockade (ICB) therapy in various malignancies, only a limited number of patients derive clinical benefits. Previous studies have suggested a link between ICB response and hypoxia signaling expression (30). However, the potential benefit of targeting hypoxia-related lncRNAs in enhancing ICB therapy remains unexplored. Using the TIDE algorithm (31) to predict responses to ICB therapy, we found that HGG patients in the high-risk group had the poor response to ICB treatment, likely due to the immunosuppressive microenvironment and immune checkpoint dysregulation. Prior research has identified several hypoxia-related lncRNAs, such as OSMR-AS1, LINC01503, and TP73-AS1, as key players in regulating the immune microenvironment (32–34). Collectively, these findings suggest that the 8-lncRNA signature could serve as potential molecular targets to reverse hypoxia-induced immunotherapy resistance in HGG.

Extensive research has identified the role of lncRNAs as biomarkers or potential therapeutic targets in cancer (11, 35). In our study, in silico analysis revealed that seven hypoxia-related lncRNAs (AP000695.4, OSMR-AS1, AC078883.3, RP11-545E17.3, LINC01057, LINC01503, TP73-AS1) were associated with high risk, while only one, LINC00672, acted as a protective lncRNA. Previous study has shown that lncRNA AP000695.4 promotes epithelial-to-mesenchymal transition in serous ovarian cancer by competitively binding miR-101-3p to regulate ZEB1 expression (36). Suppression of lncRNA AC078883.3 contributes to chemoresistance against cisplatin by freeing miR-19a from PTEN (37). LINC00672 has been implicated in p53-mediated suppression of LASP1 through its interaction with hnRNPs, indicating its potential clinical value for endometrial carcinoma chemotherapy (38). Overexpression of OSMR-AS1 (32), LINC01057 (39), LINC01503 (40), or TP73-AS1 (41) can promote HGG progression. However, no previous studies have explored the roles of these lncRNAs in hypoxia-induced immunosuppression. Our study also demonstrated that knocking down TP73-AS1, a key component of the risk signature, suppresses HGG progression. Additionally, TP73-AS1 inhibition hinders TAM recruitment and suppresses M2 polarization, suggesting its potential in TAM-targeted therapies. Further research is needed to elucidate the mechanisms by which TP73-AS1 regulates TAM infiltration and polarization.

In summary, we identified eight hypoxia-related lncRNAs with significant biological and clinical relevance, which could inform personalized therapeutic strategies. These lncRNAs may serve as indicators for HGG prognosis and responsiveness to immunotherapy. However, a limitation of our study is that certain findings were based on a retrospective review of publicly accessible datasets. Thus, further investigation is necessary to understand the molecular mechanisms by which hypoxia-related lncRNAs modulate immunosuppression. We propose that hypoxia-related lncRNAs are potential therapeutic targets in HGG.

## Data Availability

The raw data supporting the conclusions of this article will be made available by the authors, without undue reservation.
