# Supplementary material for: Profiling hypoxia signaling reveals a lncRNA signature contributing to immunosuppression in high-grade glioma
Source: Front Immunol. 2024 Oct 2;15:1471388. doi: 10.3389/fimmu.2024.1471388 (PMC11479907; doi:10.3389/fimmu.2024.1471388)
Supplement: Supplementary file 1 [file Image1.pdf]

Additional figures

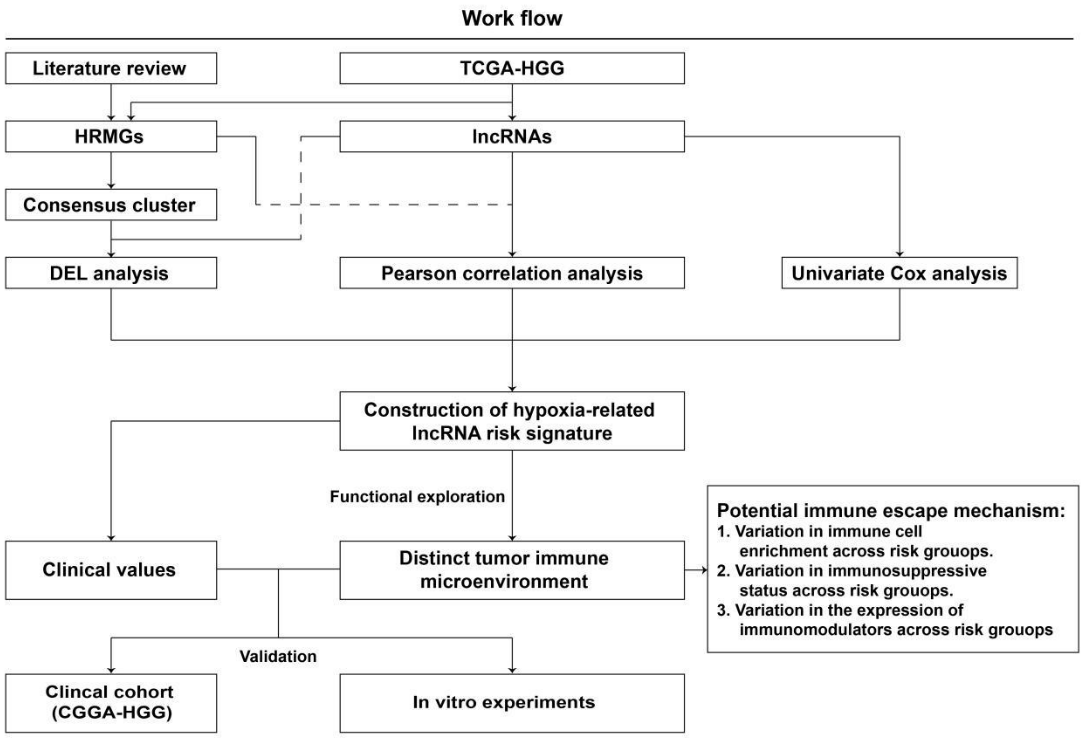

Fig. S1 Study workflow. HGG, high-grade glioma; DEL, differential expression lncRNA; HRMGs, hypoxia-related metagenes.

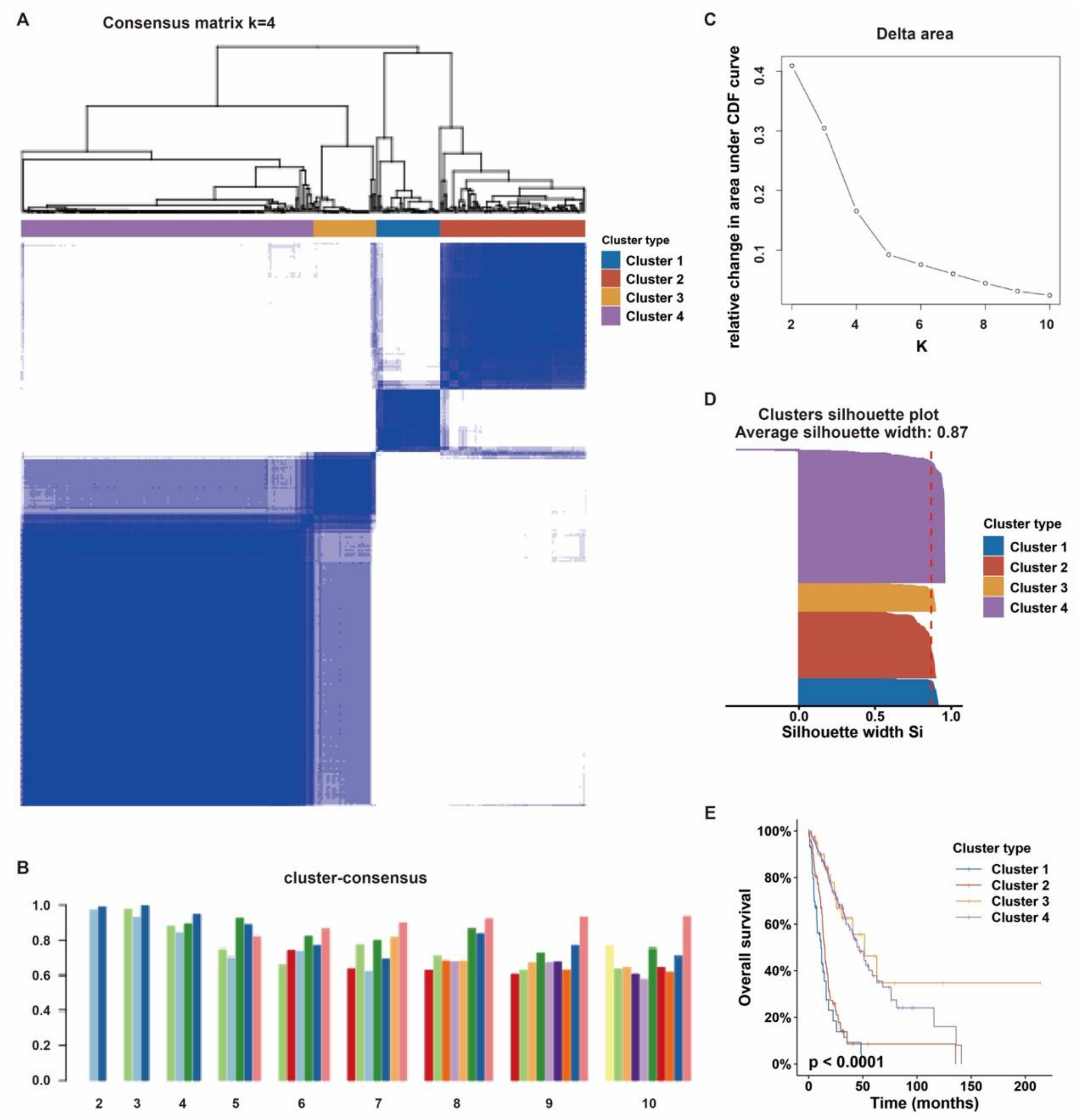

Fig. S2 Identification of hypoxia clusters in HGG. (**A**) Consensus clustering matrix of TCGA HGG samples for k = 4. (**B**) Consensus clustering stability shown in the Cluster-Consensus plot. High values indicate stability, low values indicate instability. (**C**) Delta area plot showing the relative change in area under the Consensus Cumulative Distribution Function (CDF) curve comparing k and k-1, aiding in determining optimal clustering numbers. (**D**) Silhouette analysis of hypoxia-related clustering. (**E**) KM curve showing overall survival of cases from each hypoxia cluster. p-value evaluated using the log-rank test. HGG, high-grade glioma; KM, Kaplan–Meier.

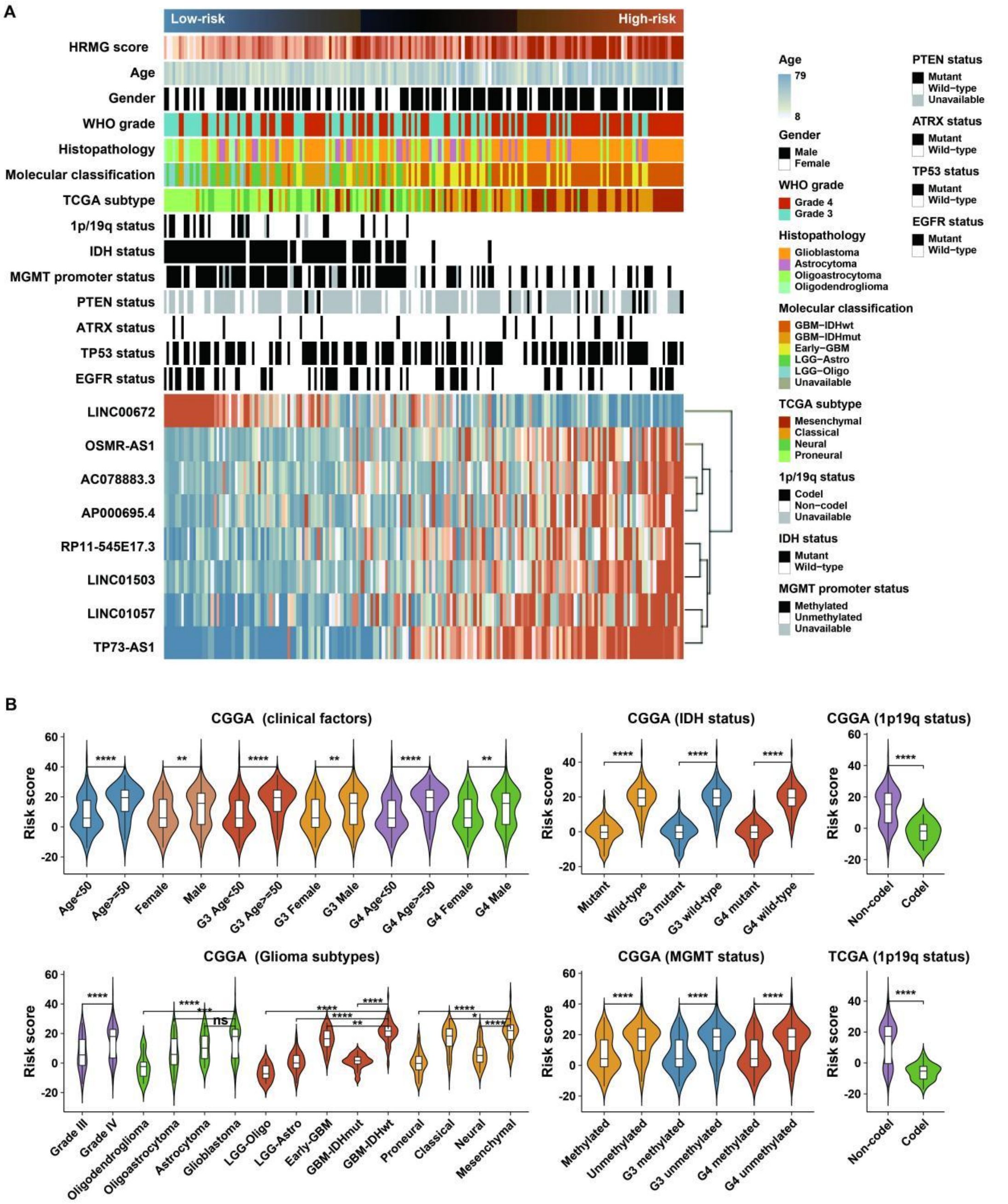

Fig. S3 Correlation between the hypoxia-related risk signature and clinical/molecular features in CGGA and TCGA. (**A**) Distribution of clinicopathological and molecular features linked to the hypoxia-related 8-lncRNA signature, sorted by ascending risk scores in HGG from the CGGA dataset. (**B**) Various distributions of risk scores were observed among clinical factors, glioma subtypes, IDH status, MGMT status, and 1p19q status. **p < 0.01; ***p < 0.001; ****p < 0.0001; ns, not significant. HGG, high-grade glioma.

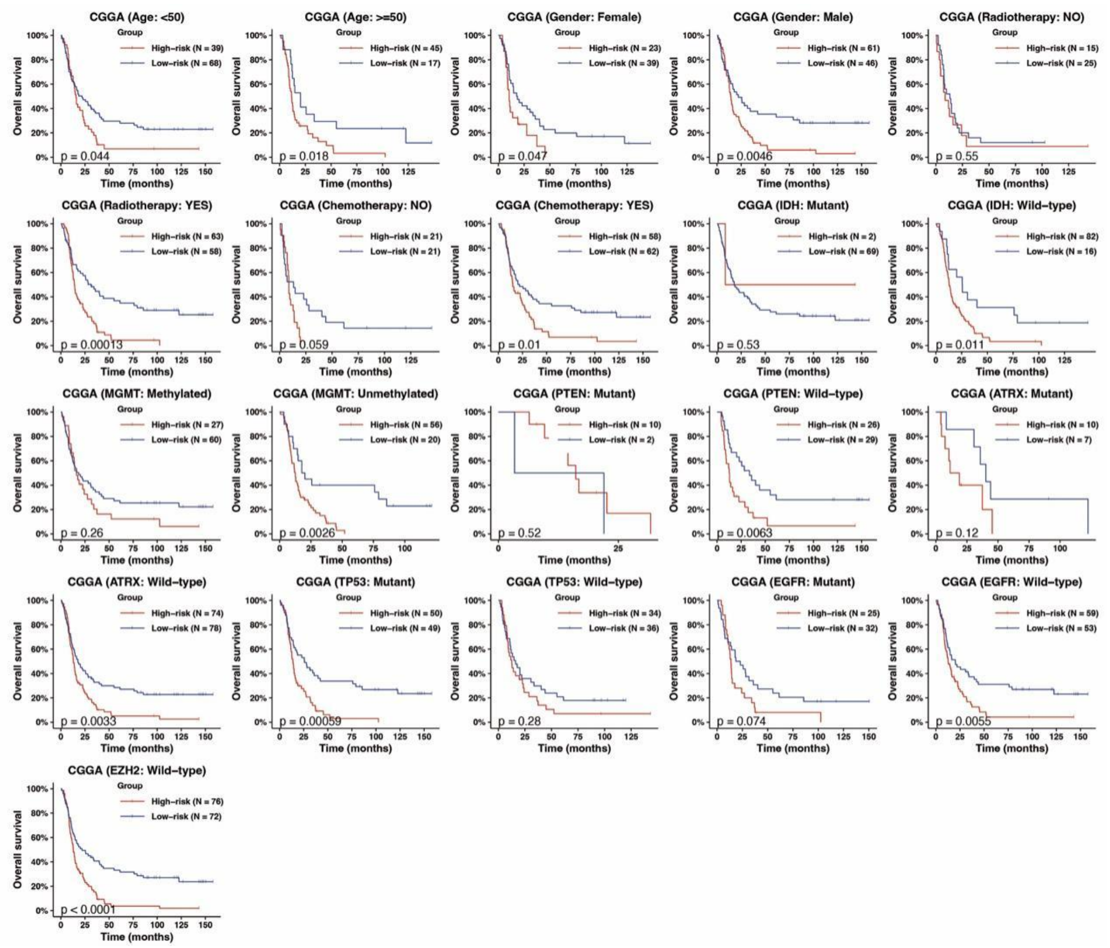

Fig. S4 Prognostic value of the hypoxia-related 8-lncRNA risk signature in stratified HGG groups from the CGGA dataset. The risk signature displayed significant prognostic value in different cohorts stratified by clinical features and key molecular events of HGG from CGGA datasets. p-values were computed using the log-rank test for trend.

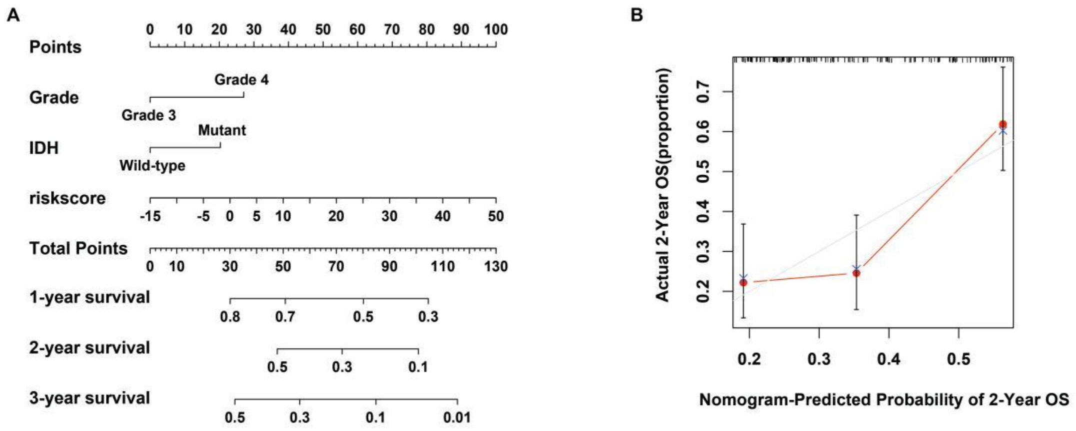

Fig. S5 Prognostic prediction of the risk signature in HGG from the CGGA dataset. Nomogram plots and calibration curves based on IDH status, WHO grade, and the lncRNA risk score were created to illustrate the prognostic prediction of the risk signature in HGG cohorts from the TCGA dataset. HGG, high-grade glioma.

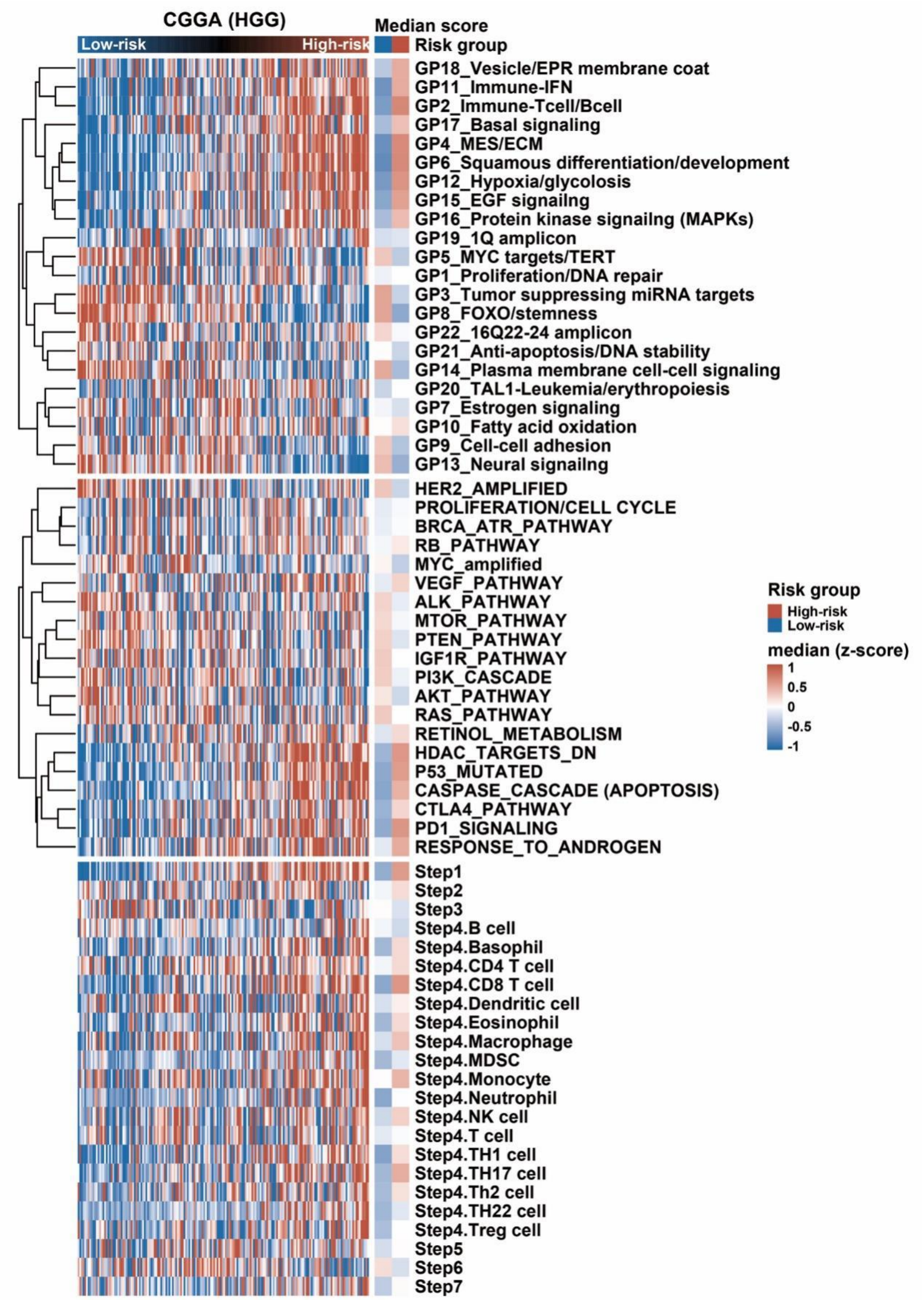

Fig. S6 High-risk HGG exhibited immunosuppressive phenotypes. The distribution of gene set scores from gene programs, pathway signatures, and tumor immunophenotype (TIP; http://biocc.hrbmu.edu.cn/TIP/) sorted by ascending risk scores in HGG from the CGGA dataset. HGG, high-grade glioma.

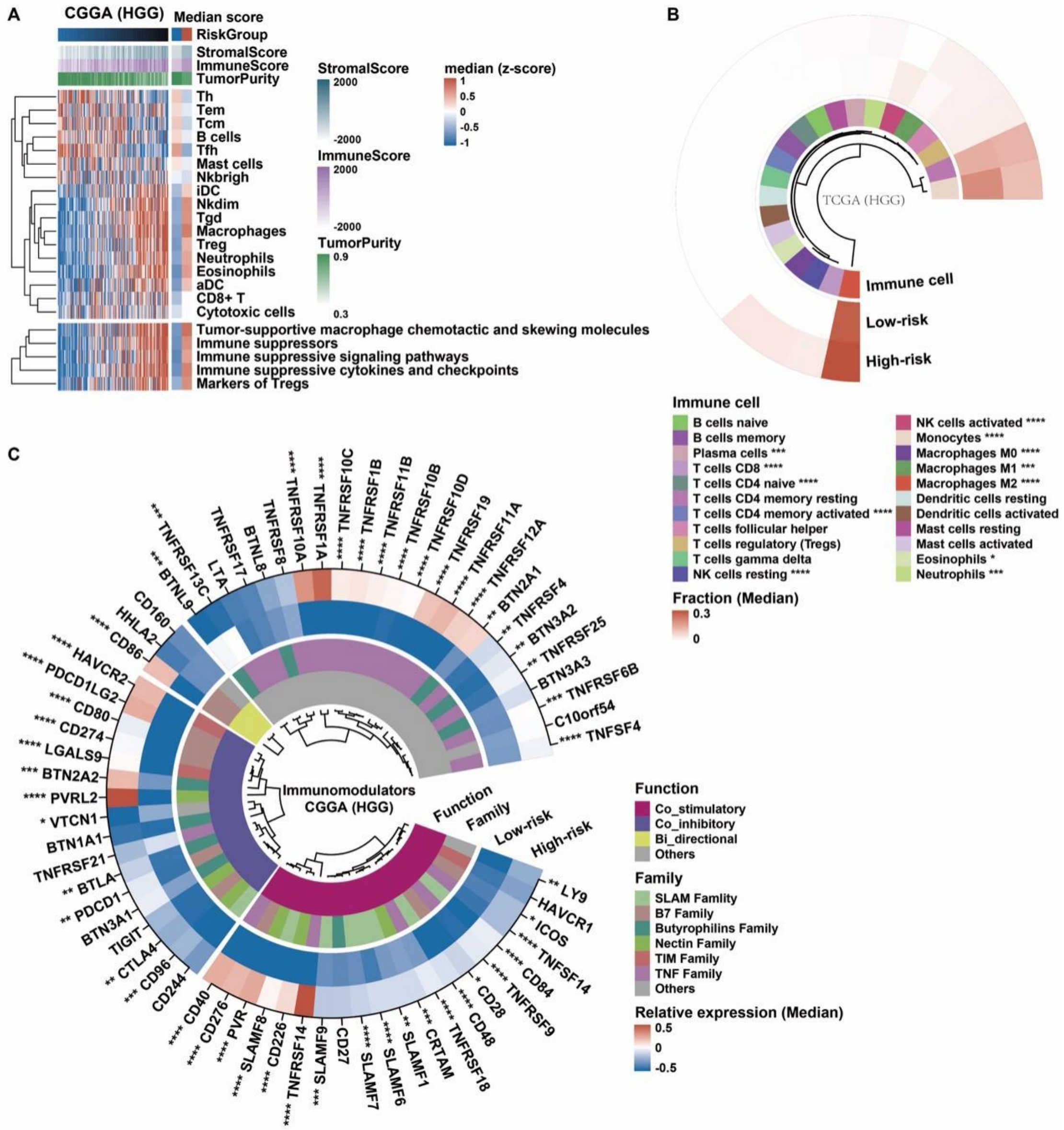

Fig. S7 Distinct immune microenvironments in HGG risk groups. (**A**) Distribution of tumor immune microenvironment-related scores sorted by ascending risk scores across HGG from the CGGA dataset. The left heatmap shows the median distribution of related scores in each risk group. (**B and C**) Differences in immune cell fractions and immunomodulator profiles between risk groups assessed by Wilcoxon test. Color intensities represent the median fraction of immune cells or the median relative expression of each immunomodulator in each risk group. *p < 0.05; **p < 0.01; ***p < 0.001; ****p < 0.0001; HGG, high-grade glioma.

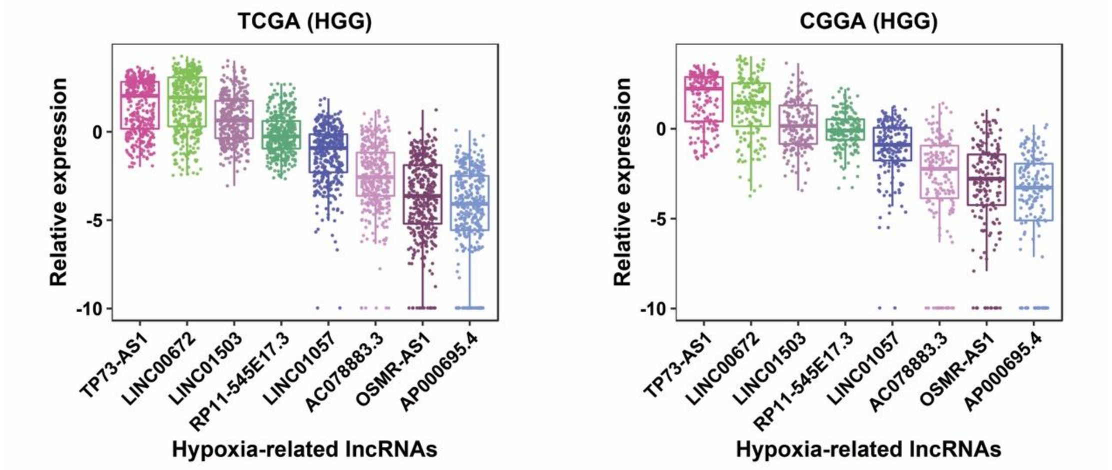

Fig. S8 Relative expression levels of eight hypoxia-related lncRNAs in HGG from the TCGA and CGGA datasets. HGG, high-grade glioma.
